# Online Registry of COVID-19–Associated Mucormycosis Cases, India, 2021

**DOI:** 10.3201/eid2711.211322

**Published:** 2021-11

**Authors:** Shitij Arora, Vagish S. Hemmige, Charuta Mandke, Mayank Chansoria, Sumit Kumar Rawat, Ameet Dravid, Yatin Sethi, Gaurav Medikeri, Sunit P. Jariwala, Yoram A. Puius, the Mycotic Infections in COVID-19 (MUNCO) Network

**Affiliations:** Albert Einstein College of Medicine, Bronx, New York, USA (S. Arora, V.S. Hemmige, S.P. Jariwala, Y.A. Puius);; Montefiore Medical Center, Bronx (S. Arora, V.S. Hemmige, S.P. Jariwala, Y.A. Puius);; HinduHrudaySamrat Balasaheb Thackarey Medical College and Dr. Rustom Narsi Cooper Municipal General Hospital, Mumbai, India (C. Mandke);; Netaji Subhash Chandra Bose Medical College, Jabalpur, India (M. Chansoria);; Bundelkhand Medical College, Sagar, India (S.K. Rawat); Noble Hospital, Pune, India (A. Dravid);; Ventakateshwar Hospital, Delhi (Y. Sethi);; HCG Comprehensive Cancer Care Hospital, Bangalore, India (G. Medikeri)

**Keywords:** COVID-19, mucormycosis, India, corticosteroids, SARS-CoV-2, respiratory infections, severe acute respiratory syndrome coronavirus 2, 2019 novel coronavirus disease, coronavirus disease, zoonoses, viruses, coronaviruses, fungus, fungal infections, fungi

## Abstract

We established an online registry of coronavirus disease–associated mucormycosis cases in India. We analyzed data from 65 cases diagnosed during April–June 2021, when the Delta variant predominated, and found that patients frequently received antibacterial drugs and zinc supplementation. Online registries rapidly provide relevant data for emerging infections.

Coronavirus disease (COVID-19)–associated mucormycosis (CAM) is an emerging systemic fungal infection caused by *Mucorales* species. Reports of CAM are increasing, especially in India, where 187 cases have been described (*1*). Rapid data collection, which can be accomplished through collaborative online registries, is essential to identifying risk factors for CAM (*2*). We analyzed characteristics of the first 65 cases logged in the Mycotic Infections in COVID-19 (MUNCO) registry in India.

We solicited registry participation through social media and contacts at hospitals in India. The study was approved by the Institutional Review Board of the Albert Einstein College of Medicine (approval no. 2021-13086) and ethics boards of the author-affiliated hospitals, where applicable. Cases were entered into a REDCap database (*3*) (https://www.covidmucor.com). CAM diagnosis was based on the judgment of the physician entering the data and not dependent on microbiological, pathologic, or radiographic findings. We had follow-up data for 53 (81.5%) patients; outcomes were defined as full recovery (no residual disease), incomplete recovery (continued treatment at day 42, interrupted treatment, palatal perforation, stroke, or paralysis), vision loss, or death. Because early treatment with orbital exenteration might prevent disease spread to the central nervous system, we did not consider vision loss to be a marker of incomplete recovery. We analyzed data using R (*4*).

The reported infections were diagnosed during April–June 2021. During this time, the B.1.617.2 lineage (Delta variant) of severe acute respiratory syndrome coronavirus 2 (SARS-CoV-2) dominated the samples sequenced by the Indian SARS-CoV-2 Genomics Consortium, constituting 58% of isolates in April, 88% in May, and 86% in June (*5*).

Most patients were male (73.8%), and most patients had diabetes (80.0%) (Table). Only 3.1% had been taking long-term corticosteroids. No patients had HIV, cancer, or history of stem cell or solid organ transplant. Among patients with available data, the median age was 56 years, median weight was 64 kg, and median hemoglobin A1c level was 7.80%. The median time between COVID-19 diagnosis and mucormycosis diagnosis was 20 days; patients had a median hospital stay of 11.0 days (Appendix Table). Only 3.1% of patients were fully vaccinated with Covishield (Oxford/AstraZeneca, https://www.astrazeneca.com) or Covaxin (Bhart Biotech, https://www.bharatbiotech.com) at the time of COVID-19 diagnosis.

**Table:** Clinical characteristics of patients in an online registry of coronavirus disease–associated mucormycosis, India, 2021*

Characteristic	No. (%)
Total	65 (100)
Sex	
M	48 (74)
F	17 (26)
Underlying conditions	
Diabetes mellitus	52 (80)
Hypertension	13 (20)
Chronic corticosteroid use	2 (3)
Asthma/COPD	1 (1.5)
Hospitalized†	54 (84)
Intensive care unit‡	15 (28)
Required surgical intervention	26 (40)
Site of infection	
Sinus	60 (92)
Eye	34 (52)
Cerebral	5 (7.7)
Gastrointestinal	5 (7.7)
Skin	1 (1.5)
Pulmonary	0
Treatment	
Steroids	53 (82)
Methylprednisolone	32 (49)
Dexamethasone	18 (28)
Prednisone	5 (8)
Budesonide	6 (9)
Steroids >10 d§	28 (61)
Antifungal medication	
Posaconazole	43 (66)
Isavuconazole	3 (5)
Amphotericin B	60 (92)
Liposomal	54 (83)
Deoxycholate	14 (22)
Lipid complex	8 (12)
Antiviral medication	
Remdesivir	31 (48)
Favipravir	18 (28)
Zinc supplementation	36 (55)
Other antimicrobial chemotherapy	
Doxycycline	30 (46)
Azithromycin	25 (38)
Ivermectin	25 (38)
No. vaccine doses	
0	56 (86)
1	7 (11)
2	2 (3)

*Totals might exceed 100% when >1 category applies. COPD, chronic obstructive pulmonary disease. †Data available for 64 patients. ‡Data available for 54 patients. §Data available for 46 patients.

COVID-19 was treated primarily with corticosteroids, remdesivir, or both. Favipravir, doxycycline, azithromycin, ivermectin, and zinc were also common treatments (Table). No patients were treated with tocilizumab.

We found that most fungal infections occurred in the sinuses or eyes (Table). Amphotericin B, posaconazole, and surgery were the most common antifungal treatments. Among 53 patients with available follow-up data at 42 days, 17 (32.1%) had an incomplete recovery, 20 (37.8%) had a full recovery, 10 (18.9%) had vision loss, and 6 (11.3%) had died (Figure).

**Figure:**
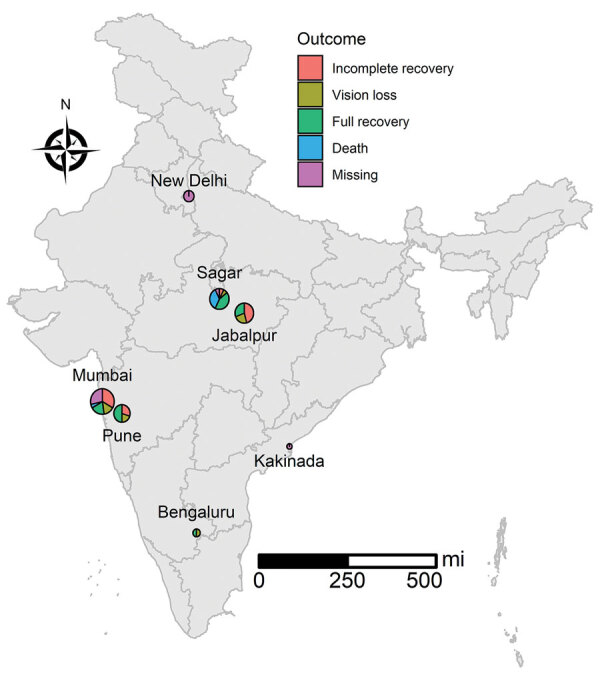
Geographic distribution of coronavirus disease–associated mucormycosis, India, 2021. Sizes of circles indicates number of cases in that area. Use of the map recognized by the government of India does not endorse the territorial claims of any specific nation.

In agreement with previous studies, we found that diabetes and steroid use were major risk factors for CAM (*1*,*6*). We also documented frequent use of antibacterial treatments, a documented risk factor for mucormycosis (*10*), for COVID-19. We found a lower death rate than previously reported (*1*); widespread awareness of CAM might have contributed to increased reporting, earlier diagnosis, and decreased steroid use for treatment of COVID-19. In total, 57% of patients received zinc supplementation, possibly because pathogenic fungi sequester zinc from host tissues. Zinc chelators inhibit the growth of some virulent fungi (*7*,*8*) and enhance the efficacy of antifungal agents against some *Mucorales* strains in vitro (*9*).

This proof-of-concept study shows that rapid, real-time data collection using online registries of CAM cases can provide clinical insights into the disease (*2*). For example, data on these 65 cases were collected in 5 days, enabled by rapid data entry and ease of use. MUNCO is especially useful for physicians in settings where electronic medical records are rarely used and patient follow-up is suboptimal. The major weakness of MUNCO is that pragmatic case definitions are based on the opinions of the clinician entering the data. This study also did not have a control group of non–COVID-19–associated mucormycosis cases, which would enable detection of specific risk factors. By August 2021, we had collected data on 693 cases, which we will soon analyze for additional risk factors associated with poor outcomes. In summary, our results show that online registries are a valuable tool to rapidly provide relevant data for real-time surveillance of emerging infections.

Appendix Additional data for online registry of COVID-19–associated mucormycosis cases, India, 2021.
