# Supplementary material for: Online Registry of COVID-19–Associated Mucormycosis Cases, India, 2021
Source: Emerg Infect Dis. 2021 Nov;27(11):2963–5. doi: 10.3201/eid2711.211322 (PMC8544967; doi:10.3201/eid2711.211322)
Supplement: Appendix — Additional data for online registry of COVID-19–associated mucormycosis cases, India, 2021. [file 21-1322-Techapp-s1.pdf]

Article DOI: https://doi.org/10.3201/eid2711.211322

Online Registry of COVID-19–Associated Mucormycosis Cases, India, 2021

Appendix

Appendix Table. Demographic characteristics of patients in an online registry of coronavirus disease–associated mucormycosis, India, 2021

Characteristic	No. patients	Value
Age, y	65	56 (45–65)
Weight, kg	58	64 (59–70)
Body mass index, kg/m^2	53	23.46 (22.38–25.80)
Hemoglobin A1c, %	47	7.80 (6.85–10.61)
Time between coronavirus disease and mucormycosis diagnoses, d	61	20 (15–25)
Duration of hospitalization, d	54	11 (7.2–15.0)

*Values are median (interquartile range).
